# Flesh flies (Diptera: Sarcophagidae) colonising large carcasses in Central Europe

**DOI:** 10.1007/s00436-015-4431-1

**Published:** 2015-04-01

**Authors:** Krzysztof Szpila, Anna Mądra, Mateusz Jarmusz, Szymon Matuszewski

**Affiliations:** 1 Chair of Ecology and Biogeography, Faculty of Biology and Environmental Protection, Nicolaus Copernicus University, Lwowska 1, 87-100 Toruń, Poland; 2 Laboratory of Criminalistics, Faculty of Law and Administration, Adam Mickiewicz University, Św. Marcin 90, 61-809 Poznań, Poland; 3 Department of Animal Taxonomy and Ecology, Faculty of Biology, Adam Mickiewicz University, Umultowska 89, 61-614 Poznań, Poland

**Keywords:** Sarcophagidae, Europe, Succession, Carrion decomposition, Forensic entomology

## Abstract

Sarcophagidae are an important element of carrion insect community. Unfortunately, results on larval and adult Sarcophagidae from forensic carrion studies are virtually absent mostly due to the taxonomic problems with species identification of females and larvae. The impact of this taxon on decomposition of large carrion has not been reliably evaluated. During several pig carcass studies in Poland, large body of data on adult and larval Sarcophagidae was collected. We determined (1) assemblages of adult flesh flies visiting pig carrion in various habitats, (2) species of flesh flies which breed in pig carcasses, and (3) temporal distribution of flesh fly larvae during decomposition. Due to species identification of complete material, including larvae, females, and males, it was possible for the first time to reliably answer several questions related to the role of Sarcophagidae in decomposition of large carrion and hence define their forensic importance. Fifteen species of flesh flies were found to visit pig carcasses, with higher diversity and abundance in grasslands as compared to forests. Sex ratio biased towards females was observed only for *Sarcophaga argyrostoma, S. caerulescens*, *S. similis* and *S. carnaria* species group. Gravid females and larvae were collected only in the case of *S. argyrostoma*, *S. caerulescens*, *S. melanura* and *S. similis. Sarcophaga caerulescens* and *S. similis* bred regularly in carcasses, while *S. argyrostoma* was recorded only occasionally. First instar larvae of flesh flies were recorded on carrion earlier or concurrently with first instar larvae of blowflies. Third instar larvae of *S. caerulescens* were usually observed before the appearance of the third instar blowfly larvae. These results contest the view that flesh flies colonise carcasses later than blowflies. *Sarcophaga caerulescens* is designated as a good candidate for a broad forensic use in Central European cases.

## Introduction

Family Sarcophagidae is a large taxon represented by more than 2600 species (Pape 1996) with Old World’s domination of a megadiverse genus *Sarcophaga* Meigen, 1826. Most flesh flies are parasites or predators with relatively small number of species with true preference for vertebrate carrion (Povolný and Verves 1997; Richet et al. 2011). Flesh flies are usually considered as a taxon of high forensic importance (e.g. Smith 1986; Byrd and Castner 2009). However, their participation in carrion insect communities was tested almost exclusively in experiments with small sized carrion (e.g. Denno and Cothran 1976; Hanski 1976, 1987; Hanski and Kuusela 1980; Kuusela and Hanski 1982; Blackith and Blackith 1990). In Europe, large vertebrate carrion, including human cadavers, attracts 33 species of Sarcophagidae, but only a few of them use this substrate for breeding (Arnaldos et al. 2004; Grassberger and Frank 2004; Matuszewski et al. 2008; Bonacci et al. 2010; Prado e Castro et al. 2010; Anton et al. 2011). Diversity and abundance of flesh flies on large carrion increase towards south of Europe with the highest values in Mediterranean countries (Arnaldos et al. 2004; Grassberger and Frank 2004; Matuszewski et al. 2008; Prado e Castro et al. 2010; Anton et al. 2011). However, successional studies concerning species identification of Sarcophagidae are rare. Additionally, most results are seriously biased due to taxonomic difficulties with female specimens identification. The female-biased sex ratio of carrion fly assemblages was reported in Calliphoridae, Muscidae, and Sarcophagidae (Martin-Vega and Baz 2013). Consequently, lack of species identification of female specimens may result in misconception on the importance of Sarcophagidae in carrion insect communities.

Diversity of adult flies gives only partial information about carrion fly community. In this context, activity of larvae is more important. Forensic entomology textbooks recommend rearing of flesh fly larvae for identification purposes (Smith 1986; Byrd and Castner 2009). Unfortunately, this method was successful (Grassberger and Frank 2004; Anton et al. 2011) only in the case of about half of the collected larvae due to the above-mentioned problems with identification of females.

An important source of information about breeding of flesh flies in large carrion are also forensic case reports. Unfortunately, cases with correct species identification of flesh flies are very rare (Povolný and Verves 1997; Benecke 1998; Draber-Mońko et al. 2009; Pohjoismäki et al. 2010; Sukontason et al. 2010; Velásquez et al. 2010; Cherix et al. 2012; Bonacci et al. 2014; Nassu et al. 2014; Vasconcelos et al. 2014). The list of species of Sarcophagidae recorded in larval stage on human corpses in Europe is surprisingly low as compared to list of species recorded during carrion experiments (6 species and 33 species). In Europe, larval *S. argyrostoma* is regularly collected on human corpses (Povolný and Verves 1997; Benecke 1998; Draber-Mońko et al. 2009; Velásquez et al. 2010; Cherix et al. 2012). Other species are recorded only occasionally and mostly in the Mediterranean countries (Velásquez et al. 2010; Bonacci et al. 2014).

Recent monograph of Richet et al. (2011), key of Szpila et al. (2015) and molecular work of Jordaens et al. (2013) made it possible to overcome the problem of species identification of European necrophagous flesh flies. Keys and illustrations published by Richet et al. (2011) and Szpila et al. (2015) made it possible to identify specimens of Sarcophagidae, including females and larvae, collected during large-scale successional experiments conducted recently in Poland (Matuszewski et al. 2008, 2010, 2014). Accordingly, it was possible for the first time to reliably answer several questions related to the role of Sarcophagidae in decomposition of large carrion and hence define their forensic importance. The aims of the present article were as follows: (1) to define assemblages of adult flesh flies on large carrion in various habitats, (2) to find out which species of flesh flies breed in large carrion, and (3) to characterise temporal distribution of flesh fly larvae during decomposition.

## Material and methods

### Field experiments

Detailed descriptions of experimental design and protocols for sampling and handling of insects are available in Matuszewski et al. (2008, 2010, 2013, 2014). Below, only the most important points are summarised.

From 2005 to 2007, 13 carcasses were exposed in pine-oak forest, 13 in alder forest, and 13 in hornbeam-oak forest. In 2011, nine carcasses were exposed in grasslands, four in ecotone at the edge of a forest, four in ecotone in birches in grasslands, five in hornbeam-oak forest, four in alder forest and four in birch forest. In 2012, all 24 carcasses were exposed in grasslands.

Adult flies were sampled manually with aerial sweep net and with pitfall traps. Larval flies were sampled with forceps and with pitfall traps. At each carcass, two traps (with 50 % ethylene glycol solution) were used. Insects were preserved in 70 % ethanol. Until about the end of active decay, insects were sampled daily, and afterwards, sampling was less frequent.

### Species identification

**Fig. 1:**
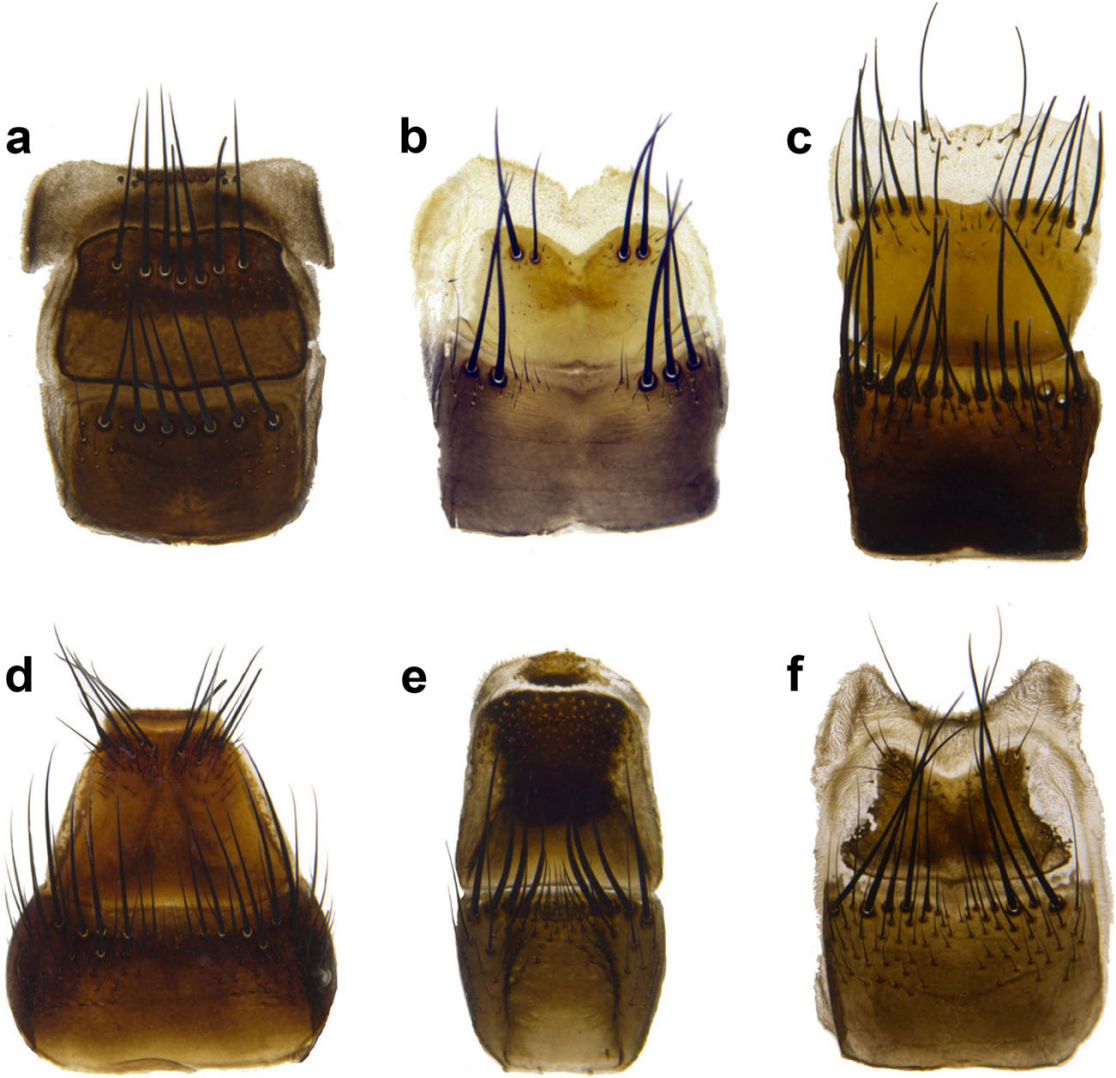
Shape of sixth and seventh sternite of female abdomen of the six species of *Sarcophaga* collected in experiments. **a** *S. albiceps*, **b** *S. argyrostoma*, **c** *S. carnaria* species group, **d** *S. caerulescens*, **e** *S. melanura* and **f** *S. similis*

Identification of males and females was made by using monographs of Pape (1987), Povolný and Verves (1997) and Richet et al. (2011). Females were identified mostly through the analysis of the shape of the sixth and seventh sternite of the ovipositor. This characteristic makes it possible to unambiguously identify females of the most important Central and Northern European necrophagous and copro-necrophagous flesh flies (i.e. *S. africa*, *S. albiceps*, *S. argyrostoma*, *S. caerulescens*, *S. melanura* and *S. similis*) (Fig. 1). Females from *S. carnaria* species group were not identified to the species level. Gravidity of females was checked by dissection of the abdomen.

Larvae were identified using key of Szpila et al. (2015) and illustrations of Richet et al. (2011).

### Statistical analysis

Significance of differences in diversity (as quantified by the number of species) and abundance of adult and larval Sarcophagidae between habitats was evaluated using the Kruskal-Wallis test. These analyses were performed only for results of 2011 experiment.

Significance of differences between flesh flies and blowflies in the preappearance interval (PAI) of first instar larvae and third instar larvae was tested using the Wilcoxon signed-rank test. These analyses were performed only for results of 2012 experiment.

The preappearance interval was defined and used throughout the manuscript as an interval from the moment of death until the appearance of first specimen of a given stage (Matuszewski and Szafałowicz 2013).

In all analyses, a 5 % level of significance was accepted. Calculations were made using Statistica 10 (StatSoft, Inc. 1984–2011).

## Results

### Species diversity and habitat preferences of Sarcophagidae

**Fig. 2:**
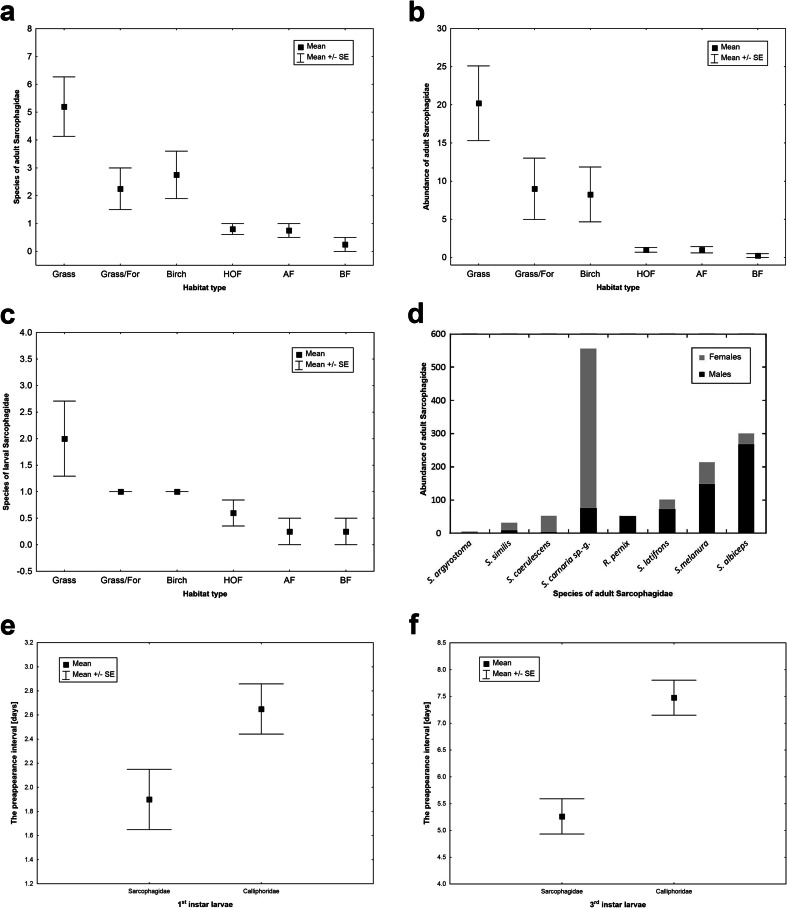
Differences between habitats in the number of species of adult (**a**) or larval (**c**) flesh flies and abundance of adult flesh flies (**b**). Sex ratio of the most abundant species (**d**). Differences between flesh flies and blowflies in the preappearance interval of the first instar larvae (**e**) and third instar larvae (**f**). *HOF* hornbeam-oak forest, *AF* alder forest, *BF* birch forest, *Grass/For* grasslands at the edge of a forest, *Birch* birches in grasslands, *Grass* grasslands

Specimens of adult flesh flies belonging to 15 species were collected during 5 years of experiments (Table 1). The most numerous were species from subfamily Sarcophaginae with 10 species of *Sarcophaga* (*sensu* Pape 1996) and one species of *Ravinia* Robineau-Desvoidy, 1863. Subfamily Paramacronychiinae was represented by *Sarcophila latifrons*, and Miltogramminae was represented by few specimens belonging to two species. The highest number of species and the largest abundance of flesh flies were recorded on carcasses in grassland and ecotone habitats (Table 1, Fig. 2a, b; Kruskal-Wallis test, number of species in adult stage: *H*_(5,26)_ = 18.3, *P* = 0.0026, abundance of adult flesh flies: *H*_(5,26)_ = 17.8, *P* = 0.0032). Adults of *S. caerulescens* and species of *S. carnaria* species group were highly regular visitors on carcasses in these habitats (Table 1). *Sarcophaga melanura* and *S. albiceps* were frequently noticed in grassland habitats (Table 1). It is also of importance that *S. latifrons* and *R. pernix* were relatively frequently collected in grassland habitats. Single specimens of *S. argyrostoma* were present on only three carcasses in grassland habitats. The number of species and their abundance was very low in forest habitats (Table 1, Figs. 2a, b). Presence of adult flesh flies was usually restricted in these habitats to a few specimens visiting occasionally only some carcasses. Species of *S. carnaria* species group and *S. caerulescens* were slightly more frequent in forest habitats.

**Table 1:** Frequency of flesh flies on pig carcasses in different habitats

Species	Habitat						
	Forest (*n* = 52)				Ecotone (*n* = 8)		Open (*n* = 33)
	POF (*n* = 13)	AF (*n* = 17)	HOF (*n* = 18)	BF (*n* = 4)	Birch (*n* = 4)	Grass/For (*n* = 4)	Grassland
*Metopia campestris* (Fallén, 1810)			A (*n* = 1)				
*Miltogramma germari* (Meigen, 1824)							A (*n* = 1)
*Sarcophila latifrons* (Fallén, 1817)					A (*n* = 2)	A (*n* = 1)	A (*n* = 20)
*Ravinia pernix* (Harris, 1780)		A (*n* = 3)			A (*n* = 2)		A (*n* = 14)
*Sarcophaga albiceps* (Meigen, 1826)	A (*n* = 1)	A (*n* = 1)		A (*n* = 1)		A (*n* = 1)	A (*n* = 20)
*Sarcophaga aratrix* (Pandellé, 1896)		A (*n* = 1)					A (*n* = 2)
*Sarcophaga argyrostoma* (Robineau-Desvoidy, 1830)							A (*n* = 3) L (*n* = 3)
*Sarcophaga caerulescens* (Zetterstedt, 1838)	A (*n* = 1) L (*n* = 1)	A (*n* = 2)	A (*n* = 5) L (*n* = 5)	A (*n* = 2) L (*n* = 2)	A (*n* = 4) L (*n* = 4)	A (*n* = 4) L (*n* = 4)	A (*n* = 31) L (*n* = 31)
*Sarcophaga incisilobata* (Pandellé, 1896)							A (*n* = 6)
*Sarcophaga melanura* (Meigen, 1826)					A (*n* = 1)	A (*n* = 1)	A (*n* = 26) L (*n* = 5)
*Sarcophaga similis* (Meade, 1876)							A (*n* = 20) L (*n* = 19)
*Sarcophaga carnaria* species group							
*S. carnaria* (Linnaeus, 1758)			A (*n* = 1)		A (*n* = 2)	A (*n* = 1)	A (*n* = 17)
*S. lehmanni* Mueller, 1922							A (*n* = 8)
*S. subvicina* Rohdendorf, 1937	A (*n* = 1)	A (*n* = 1)					A (*n* = 5)
*S. variegata* (Scopoli, 1763)	A (*n* = 1)	A (*n* = 3)	A (*n* = 1)			A (*n* = 1)	A (*n* = 10)
Females	A (*n* = 4)	A (*n* = 5)	A (*n* = 6)	A (*n* = 2)	A (*n* = 6)		A (*n* = 30)

*POF* pine-oak forest, *AF* alder forest, *HOF* hornbeam-oak forest, *BF* birch forest, *Birch* young birch stand in grassland, *Grass/For* edge of grassland and forest, *n* number of carcasses exposed (headline), visited (for adults, A) and colonised (for larvae, L)

Only four species of flesh flies were recorded on carcasses in larval stage (Table 1). Regular colonisation occurred just in open and ecotone habitats (Table 1, Fig. 2c; Kruskal-Wallis test, number of species in larval stage: *H*_(5,26)_ = 10.8, *P* = 0.056). Larvae of *S. caerulescens* were present on most of the carcasses exposed in grasslands and ecotones. In grassland habitats, larvae of *S. similis* were also frequent (Table 1). Larvae of other species (*S. argyrostoma*, *S. melanura*) were collected occasionally and exclusively in open habitats. Forest carcasses were colonised only few times by a low number of larvae of *S. caerulescens*.

### Sex ratio of adult flies

The overwhelming predominance of females was noticed mostly due to a high abundance of species representing *S. carnaria* species group (Fig. 2d). A high proportion of females were also recorded in the case of *S. argyrostoma*, *S. caerulescens* and *S. similis*. Other species were represented mostly by males (Fig. 2d). Gravid females were collected only in the case of *S. argyrostoma*, *S. caerulescens*, *S. melanura* and *S. similis*.

### Preappearance interval of larvae

The first instar larvae of flesh flies were firstly noticed usually on the second to fourth day after carcass exposure, rarely later (Fig. 2e). The difference in PAI of the first instar larvae between flesh flies and blowflies was about 1 day, with high statistical significance (Wilcoxon test, *Z* = 3.2, *P* = 0.0014, Fig. 2e). Third instar larvae of flesh flies were not abundant but easily recognisable because of their large size. The first appearance of the third instar larvae of dominant flesh fly species (i.e. *S. caerulescens*) varied from fifth to tenth day after carcass exposure (Fig. 2f). PAI of the third instar larvae of blowflies was apparently longer (by about 2 days), with high statistical significance (Wilcoxon test, *Z* = 3.8, *P* = 0.0002, Fig. 2f). Larvae of *S. melanura* were always collected on carcasses in advanced decomposition.

## Discussion

### Species diversity and habitat preferences of Sarcophagidae

The number of recorded species (i.e. 15) is surprisingly low in relation to biodiversity of flesh flies in Central Europe (Povolný and Verves 1997). Additionally, presence of two species of Miltogramminae represented by just single specimens was clearly accidental. Some species of Miltogramminae are attracted to carrion and are able to breed in this medium (Povolný and Verves 1997; Szpila et al. 2010; Valdés-Perezgasga et al. 2010: “*Anicia* sp.”, incorrect identification of *Eumacronychia* sp.), but both *Metopia campestris* and *Miltogramma germari* are well known as kleptoparasites of Hymenoptera in soil nests. Fifteen species constitute 12.3 % of total number of Sarcophagidae known from Poland (Draber-Mońko 2007; Szpila 2013) and are substantially lower as compared to species lists from large carrion experiments in south of Europe (Arnaldos et al. 2004; Prado e Castro et al. 2010) or in New World tropics (Rosa et al. 2011). However, almost all European ubiquitous species (except *S. africa*) were recorded. Absence of *S. africa*, frequently mentioned as species of forensic importance (e.g. Smith 1986; Byrd and Castner 2009), may be explained by habitat preferences, as it is strongly synanthropic in Central Europe (Povolný and Verves 1997; Fremdt and Amendt 2014). *Sarcophaga argyrostoma* has similar habitat preferences (Fremdt and Amendt 2014) and is well known as regular coloniser of large carrion in European anthropogenic habitats and that may explain its minimal abundance during current study (Povolný and Verves 1997; Benecke 1998; Grassberger and Frank 2004; Draber-Mońko et al. 2009; Anton et al. 2011; Cherix et al. 2012). All species frequently recorded in present experiments (i.e. *S. albiceps*, *S. caerulescens*, *S. carnaria* species group, *S. melanura*, *S. similis*, *R. pernix* and *Sarcophila latifrons*) show clear preference towards open habitats with decreasing abundance from grasslands through ecotones to forests. Present results confirm broad habitat preferences of *S. caerulescens*, already illustrated by Cherix et al. (2012) and Matuszewski et al. (2013). This species, a dominant coloniser of pig carcasses in our experiments, was previously characterised by Pape (1987) and Pohjoismäki et al. (2010) as preferring “shaded semi-woodland habitats”. Matuszewski et al. (2013) demonstrated that *S. caerulescens* may frequently colonise carrion also in open habitats. Pape (1987) and Pohjoismäki et al. (2010) cited as the source of their view results of ecological experiment realised in Finland (Hanski and Kuusela 1980; Kuusela and Hanski 1982). This experiment was spatially restricted to just one type of habitat, which may be described as an ecotone or a forest in the early stage of succession. In other experiment (Hanski 1976), where various habitats were studied, *S. caerulescens* (and similarly *S. similis*) colonised baits exclusively in “open field with low grass” 100 m from the forest edge. It is in line with our results and conclusively demonstrates that an open-sunny exposure of carrion (e.g. in grassland) cannot be treated as an obstacle for *S. caerulescens* colonisation. Regular colonisation of carcasses confirms serious forensic importance of this fly species, which was suggested by recent case papers (Pohjoismäki et al. 2010; Cherix et al. 2012). This argumentation applies similarly to *S. similis*, incidentally collected as larvae on human dead bodies (Chigusa et al. 2006; Cherix et al. 2012). The high frequency and abundance of adults from species representing *S. carnaria* species group need some explanation. It is generally accepted that these flies are predators of living earthworms (Richet et al. 2011). They may develop on vertebrate remains in laboratory conditions (Richet et al. 2011), but species of *S. carnaria* species group were never reliably recorded in larval stage on experimental forensic carcasses or case cadavers (Povolný and Verves 1997). In the current material, despite the numerous presence of adult flies, larvae representing *S. carnaria* species group were not found. This finding confirms low attractiveness of carrion as breeding medium for larvae of these species. The first breeding record of *S. melanura* on large carrion is also of importance. This species is characterised as a synanthropic fly, its larvae may develop in various substrates and it is suspected of facultative predation on other fly larvae (Pape 1987; Povolný and Verves 1997; Richet et al. 2011). Our findings may support this statement. Larvae of *S. melanura* were always collected on carcasses in advanced decomposition, when tissues suitable for larvae of necrophagous blowflies or flesh flies were already depleted.

### Sex ratio of adult flies

Recent results on sex ratio of higher flies visiting carrion baits indicate significantly higher abundance of females in most tested species of Calliphoridae, Muscidae and Sarcophagidae (Martin-Vega and Baz 2013). Present results follow this pattern with reference to *S. argyrostoma*, *S. caerulescens*, *S. similis* and *S. carnaria* species group. For the other species, sex ratio was shifted towards higher abundance of males, with the highest domination of males in *R. pernix*. Presence of numerous specimens of females of *S. carnaria* species group may be explained by feeding on highly nutritious products of carrion decomposition. Phenomenon of frequent visiting of carrion by females of non-necrophagous higher flies is known at least for genus *Pollenia* Robineau-Desvoidy, 1830 (Martin-Vega and Baz 2013; Szpila et al. in prep.). In both cases, females probably complete on carrion a high-protein meal which is necessary for development of embryos and that is a typical behaviour also for necrophagous species (e.g. Wolff and Hansson 2005).

Male-biased abundance of other species may be explained by mating behaviour. In such cases, carrion is treated as a swarming point of males waiting in aggregation for females (Martin-Vega and Baz 2013).

### Preappearance interval of larvae

Studies of carrion colonisation by flesh flies were conducted exclusively on small carcasses (Denno and Cothran 1976; Hanski 1976, 1987; Hanski and Kuusela 1980; Kuusela and Hanski 1982; Blackith and Blackith 1990). Ecological experiments indicate that flesh flies are generally weak competitors in comparison to blowflies (Denno and Cothran 1976; Hanski 1987). Flesh flies colonised only few experimental carcasses or traps, and their presence in habitats was positively correlated with high patchiness of carrion resources (Hanski 1987). Hanski (1987) characterised necrophagous species of *Sarcophaga* as uncommon but consistently present in the natural carrion communities. Our findings do not fully support the statement about scarce presence of flesh flies in carrion communities for grassland and ecotone habitats, where larvae of *S. caerulescens* colonised almost all exposed carcasses. Denno and Cothran (1976) described life-history strategy of Sarcophagidae as a production of few offspring which rapidly utilise fresh carrion. It corresponds well with currently demonstrated early colonisation and rapid development of *S. caerulescens* larvae. These elements of life strategy seem to be an effective response of flesh flies to the pressure of blowflies. Unfortunately, it was not possible to check if early instar larvae of *S. caerulescens* may aggressively kill larvae of blowflies, what is a known phenomenon for *S. aratrix*—a species colonising small carrion (Blackith and Blackith 1984).

## Conclusions and further research

In Central Europe, necrophagous flesh flies are a regular and constant element of large carrion insect communities in open habitats and ecotones. Carrion in forests is only occasionally visited and colonised by small subset of species. There is a large difference in dominant flesh fly species between urban or rural habitats and semi-natural habitats. In semi-natural habitats, *S. argyrostoma* is replaced by *S. caerulescens*. The diversity of flesh flies visiting and colonising large carrion in Central Europe is low. Present results confirm forensic importance of *S. caerulescens* and *S. similis*. Particularly, *S. caerulescens* is a good candidate for a broad forensic use in Central European cases. Its advantages are as follows: (1) regular occurrence on carrion, (2) early colonisation, (3) rapid development, (4) large size favouring it to be collected in forensic cases, (5) presence in ecotones which are potentially attractive habitats for concealing of human dead bodies, (6) indoor colonisation of cadavers (Pohjoismäki et al. 2010; Cherix et al. 2012) and (7) easy identification of all developmental stages (Pape 1987; Povolný and Verves 1997; Pohjoismäki et al. 2010; Richet et al. 2011; Jordaens et al. 2013; Szpila et al. 2015). Unfortunately, reliable developmental data are still absent, which limits the broad use of *S. caerulescens* in casework. Future research should therefore concentrate on the development of *S. caerulescens* and *S. similis*. Other important issue for future forensically oriented studies is an extension of successional studies in urban habitats (including indoor locations), as well as more thorough studies of necrophagous flesh fly communities in Mediterranean region. Some interesting results referring to this issue were recently published (Baz et al. 2015).

## References

[CR1] Anton E, Niederegger S, Beutel RG (2011). Beetles and flies collected on pig carrion in an experimental setting in Thuringia and their forensic implications. Med Vet Entomol.

[CR2] Arnaldos MI, Romera E, Presa JJ, Luna A, Garcia MD (2004). Studies on seasonal arthropod succession on carrion in the southeastern Iberian Peninsula. Int J Legal Med.

[CR3] Baz A, Botias C, Martin-Vega D, Cifrian B, Diaz-Aranda LM (2015) Preliminary data on carrion insects in urban (indoor and outdoor) and periurban environments in central Spain. Forensic Sci Int 248:41–47 10.1016/j.forsciint.2014.12.012 25594690

[CR4] Benecke M (1998). Six forensic entomology cases: description and commentary. J Forensic Sci.

[CR5] Blackith RE, Blackith RM (1984). Larval aggression in Irish flesh-flies (Diptera: Sarcophagidae). Irish Nat J.

[CR6] Blackith RE, Blackith RM (1990). Insect infestations of small corpses. J Nat Hist.

[CR7] Bonacci T, Brandmayr P, Greco S, Tersaruolo C, Vercillo V, Brandmayr TZ (2010). A preliminary investigation of insect succession on carrion in Calabria (southern Italy). Terr Arthropod Rev.

[CR8] Bonacci T, Greco S, Cavalcanti B, Brandmayr P, Vercillo V (2014). The flesh fly *Sarcophaga* (*Liopygia*) *crassipalpis* Macquart, 1839 as an invader of a corpse in Calabria (Southern Italy). J Forensic Sci Crim.

[CR9] Byrd JH, Castner JL (2009). Forensic entomology: the utility of arthropods in legal investigations.

[CR10] Cherix D, Wyss C, Pape T (2012). Occurrences of flesh flies (Diptera: Sarcophagidae) on human cadavers in Switzerland, and their importance as forensic indicators. Forensic Sci Int.

[CR11] Chigusa Y, Kurahashi H, Kanasugi T, Ishii K, Kirinoki M, Hayashi-Kato N, Tokudome S, Matsuda H (2006) The achievements of forensic entomology, Japan. Abstracts of 6th International Congress of Dipterology, Fukuoka, 44 p

[CR12] Denno RF, Cothran WR (1976). Competitive interactions and ecological strategies of Sarcophagid and Calliphorid flies inhabiting rabbit carrion. Ann Entomol Soc Am.

[CR13] Draber-Mońko A, Bogdanowicz W, Chudzicka E, Pilipiuk I, Skibińska E (2007). Sarcophagidae. Fauna of Poland. Characteristics and checklist of species.

[CR14] Draber-Mońko A, Malewski T, Pomorski J, Łoś M, Ślipiński P (2009). On the morphology and mitochondrial DNA barcoding of the flesh fly *Sarcophaga* (*Liopygia*) *argyrostoma* (Robineau-Desvoidy, 1830) (Diptera: Sarcophagidae)—an important species in forensic entomology. Ann Zool.

[CR15] Fremdt H, Amendt J (2014). Species composition of forensically important blow flies (Diptera: Calliphoridae) and flesh flies (Diptera: Sarcophagidae) through space and time. Forensic Sci Int.

[CR16] Grassberger M, Frank C (2004). Initial study of arthropod succession on pig carrion in a Central European urban habitat. J Med Entomol.

[CR17] Hanski I (1976). Breeding experiments with carrion flies (Diptera) in natural conditions. Ann Entomol Fenn.

[CR18] Hanski I (1987). Carrion fly community dynamics: patchiness, seasonality and coexistence. Ecol Entomol.

[CR19] Hanski I, Kuusela S (1980). The structure of carrion fly communities: differences in breeding seasons. Ann Zool Fenn.

[CR20] Jordaens K, Sonet G, Richet R, Dupont E, Braet Y, Desmyter S (2013). Identification of forensically important *Sarcophaga* species (Diptera: Sarcophagidae) using the mitochondrial *COI* gene. Int J Legal Med.

[CR21] Kuusela S, Hanski I (1982). The structure of carrion fly communities: the size and the type of carrion. Ecography.

[CR22] Martin-Vega D, Baz A (2013). Sex-biased captures of sarcosaprophagous Diptera in carrion-baited traps. J Insect Sci.

[CR23] Matuszewski S, Szafałowicz M (2013). Temperature-dependent appearance of forensically useful beetles on carcasses. Forensic Sci Int.

[CR24] Matuszewski S, Bajerlein D, Konwerski S, Szpila K (2008). An initial study of insect succession and carrion decomposition in various forest habitats of Central Europe. Forensic Sci Int.

[CR25] Matuszewski S, Bajerlein D, Konwerski S, Szpila K (2010). Insect succession and carrion decomposition in selected forests of Central Europe. Part 2: composition and residency patterns of carrion fauna. Forensic Sci Int.

[CR26] Matuszewski S, Szafałowicz M, Jarmusz M (2013). Insects colonising carcasses in open and forest habitats of Central Europe: search for indicators of corpse relocation. Forensic Sci Int.

[CR27] Matuszewski S, Konwerski S, Frątczak K, Szafałowicz M (2014). Effect of body mass and clothing on decomposition of pig carcasses. Int J Legal Med.

[CR28] Nassu MP, Thyssen PJ, Linhares AX (2014). Developmental rate of immatures of two fly species of forensic importance: *Sarcophaga* (*Liopygia*) *ruficornis* and *Microcerella halli* (Diptera: Sarcophagidae). Parasitol Res.

[CR29] Pape T (1987) The Sarcophagidae (Diptera) of Fennoscandia and Denmark. Fauna Entomologica Scandinavica (Book 19). Brill Academic Pub, Leiden-Copenhagen

[CR30] Pape T (1996). Catalogue of the Sarcophagidae of the world (Insecta: Diptera). Mem Entomol Int.

[CR31] Pohjoismäki JL, Karhunen PJ, Goebeler S, Saukko P, Sääksjärvi IE (2010). Indoors forensic entomology: colonization of human remains in closed environments by specific species of sarcosaprophagous flies. Forensic Sci Int.

[CR32] Povolný D, Verves Y (1997). The flesh-flies of Central Europe.

[CR33] Prado e Castro C, Garcia MD, Arnaldos MI, Gonzalez-Mora D (2010). Sarcophagidae (Diptera) attracted to piglet carcasses including new records for Portuguese fauna. Graellsia.

[CR34] Richet R, Blackith RM, Pape T (2011). *Sarcophaga* of France (Diptera: Sarcophagidae).

[CR35] Rosa TA, Babata MLY, de Souza CM, de Souza D, de Mello-Patiu CA, Vaz-de-Mello FZ, Mendes J (2011). Arthropods associated with pig carrion in two vegetation profiles of Cerrado in the State of Minas Gerais, Brazil. Rev Bras Entomol.

[CR36] Smith KGV (1986). A manual of forensic entomology.

[CR37] Sukontason K, Bunchu N, Chaiwong T, Moophayak K, Sukontason KL (2010). Forensically important flesh fly species in Thailand: morphology and developmental rate. Parasitol Res.

[CR38] Szpila K (2013). *Metopodia pilicornis* (Pandelle, 1895) (Diptera, Sarcophagidae)—new to the polish fauna. Dipteron.

[CR39] Szpila K, Voss JG, Pape T (2010). A new dipteran forensic indicator in buried bodies. Med Vet Entomol.

[CR40] Szpila K, Richet R, Pape T (2015) Third instar larvae of flesh flies (Diptera: Sarcophagidae) of forensic importance – critical review of characters and key for European species. Parasitol Res doi:10.1007/s00436-015-4421-3 10.1007/s00436-015-4421-3 PMC4430590 25823900

[CR41] Valdés-Perezgasga MT, Sanchez-Ramos FJ, Garcia-Martinez O, Anderson GS (2010). Arthropods of forensic importance on pig carrion in the Coahuilan Semidesert, Mexico. J Forensic Sci.

[CR42] Vasconcelos SD, Soares TF, Costa DL (2014). Multiple colonization of a cadaver by insects in an indoor environment: first record of *Fannia trimaculata* (Diptera: Fanniidae) and *Peckia* (*Peckia*) *chrysostoma* (Sarcophagidae) as colonizers of a human corpse. Int J Legal Med.

[CR43] Velásquez Y, Magaña C, Martínez‐Sánchez A, Rojo S (2010). Diptera of forensic importance in the Iberian Peninsula: larval identification key. Med Vet Entomol.

[CR44] Wolff H, Hansson C (2005). Rearing *Lucilia sericata* for chronic ulcer treatment—an improved method. Acta Derm Venereol.

